# Recognition Memory for Colored and Black-and-White Scenes in Normal and Color Deficient Observers (Dichromats)

**DOI:** 10.1371/journal.pone.0098757

**Published:** 2014-05-30

**Authors:** Serge Brédart, Alyssa Cornet, Jean-Marie Rakic

**Affiliations:** 1 Department of Psychology, University of Liège, Liège, Belgium; 2 Department of Ophthalmology, University Hospital, Liège, Belgium; University of L’Aquila, Italy

## Abstract

Color deficient (dichromat) and normal observers’ recognition memory for colored and black-and-white natural scenes was evaluated through several parameters: the rate of recognition, discrimination (*A’*), response bias (*B”D*), response confidence, and the proportion of conscious recollections (Remember responses) among hits. At the encoding phase, 36 images of natural scenes were each presented for 1 sec. Half of the images were shown in color and half in black-and-white. At the recognition phase, these 36 pictures were intermixed with 36 new images. The participants’ task was to indicate whether an image had been presented or not at the encoding phase, to rate their level of confidence in his her/his response, and in the case of a positive response, to classify the response as a Remember, a Know or a Guess response. Results indicated that accuracy, response discrimination, response bias and confidence ratings were higher for colored than for black-and-white images; this advantage for colored images was similar in both groups of participants. Rates of Remember responses were not higher for colored images than for black-and-white ones, whatever the group. However, interestingly, Remember responses were significantly more often based on color information for colored than for black-and-white images in normal observers only, not in dichromats.

## Introduction

The present study was designed to evaluate whether normal observers and dichromats differ in their memory for colored and black-and-white scenes by using different dependent variables such as corrected rates of recognition, discrimination, bias, confidence ratings and state of consciousness associated with recognition.

Normal color vision results from light absorption by three types of pigment located in the cones on the retina. The short-wave (SW), middle-wave (MW) and long-wave (LW) photopigments in the cones are maximally sensitive in short- (about 420 nm), medium- (about 530 nm) and long-wavelengths (about 560 nm) respectively [1], [2]. Normal light absorption curves for the three cones are required for normal trichromatic vision. The most common inherited color vision deficiency, often called red-green color vision deficiency, is experienced by 8% of men and 0.5% of women [2]. This red-green “color blindness” includes different dichromat and anomalous trichromat conditions. Dichromacy is a color vision deficiency in which one kind of cones is absent or does not function. Protanopia is due to the absence of LW pigments while deuteranopia is the consequence of the absence of MW pigments. These two red-green color vision deficiencies respectively affect 1% and 1.4% of men. Anomalous trichromacy occurs when the spectral sensitivity of one pigment is altered. Protanomaly is due to the alteration of the LW pigment while deuteranomaly is due to the alteration of the MW pigment. Protanomaly and deuteranomaly respectively affect 1% and 4.6% of men.

Cognitive investigation of dichromats’ color naming and categorization abilities has produced interesting results [3], [4]. It has been demonstrated that although dichromats most often confuse stimuli when arranging them along the red-green color dimension, they are able to name these stimuli in relatively good agreement with normal observers (normal trichromats; NTs). For instance, when administered the Farnsworth D-15 test, dichromats may place cap #2 (perceived as blue-green by NTs) next to cap #13 (perceived as violet-blue by NTs) while correctly naming these two caps “blue gray” and “lavender” respectively [6]. Another study indicated that dichromats’ performance was significantly better (i.e. more similar to normal observers’ performance) in a color-naming task using eight basic color terms (BCT: red, green, yellow, blue, brown, pink, orange, and purple) consisting of naming color samples than it was in a free sorting task [5]. Recently, Lillo et al. [7] reported that when the participants’ task was to select the best example of a given BCT, there was a significant difference between dichromats and NTs only for derived colors (brown, purple, orange, pink and gray) but not for primary colors (red, green, yellow, blue, black and white). Such results support the notion that the dichromats’ lexical representation of color names roughly matches that of normal observers. Interestingly this lexico-semantic representation seems to be dissociated from the protanopes’ and deuteranopes’ perceptual representation of colors [5], [6].

In comparison with color categorization and naming, there are relatively few studies of color memory in dichromats. Several studies have shown that color enhances visual recognition memory for natural scenes in normal observers [8]–[10]. To our knowledge, only one study has assessed the contribution of color to episodic recognition of natural scenes in dichromats [11]. This important study revealed that recognition memory performance is better for colored than for black-and-white pictures in dichromats too. Moreover the size of the advantage of colored pictures over black-and-white pictures is the same for dichromats as for NTs. In the Gegenfurtner et al. [11] study, the dependent measure analyzed was the hit rate, i.e. a measure of recognition accuracy. In order to make more complete the comparison between NTs and dichromats’ recognition memory for colored and black-and-white scenes, it is necessary to examine other features of recognition such as response discrimination, response bias, response confidence and the phenomenal subjective experience that accompanies recognition. Indeed, even if NTs’ perception of more subtle chromatic information does not improve their scene memory performance in comparison with dichromats, it could be that this more precise perception of colors is sufficient to influence the level of confidence and the state of consciousness associated with the recognition of a scene. Two different states of consciousness may accompany recognition i.e., *remembering* when the participant can consciously recollect something he or she experienced when that scene was initially encoded (Remember response; e.g., “When I saw that picture I thought of my last visit to my father’s house”) and *knowing* when the participant has no recollection but just a strong feeling of familiarity toward that item encountered in the experimental context (Know response; for the Remember/Know distinction see [12], [13]). The content of participants’ justifications for their Remember responses was analyzed to check whether color information was included in recollections. Because of their difficulties in perceiving colors, it is possible that dichromats would base their Remember responses on color information less often compared with NTs.

## Method

### Ethics Statement

This study was approved by the Ethics Committee of the Faculty of Psychology and Education of the University of Liège. All participants gave written informed their consent prior to participation.

### Participants

Color deficient participants were recruited through advertisements sent by email to the University of Liège community. In the first session, 30 color deficient observers (29 men) and 32 NTs (30 men) were selected on the basis of their performance in the Farnsworth D-15 test. This session also included the administration of the experimental task. During the second session, participants’ visual acuity was measured, and color vision was more precisely assessed with an anomaloscope (Tomey F-2 apparatus). The final sample included 24 dichromats (13 deuteranopes and 11 protanopes) and 22 NTs. The 16 participants who were excluded from the study consisted of: 9 participants with a protanomaly, 2 participants with a deuteranomaly, 2 participants for whom it was impossible to reach a clear diagnosis and 3 participants who did not attend the second session. All the included participants had normal or corrected to normal visual acuity. Dichromats ranged in age from 19 to 56 years (mean age = 28.88 years, *SD* = 10.45), NTs ranged from 18 to 51 years (mean age = 28.09 years, *SD* = 10.14). The two groups did not significantly differ in age, *t* (44) <1. In addition, there was no significant difference between the groups in terms of educational level measured by the number of years of study completed, *t* (44) <1; in dichromats *m* = 16.38 (*SD* = 2.95), in NTs *m* = 16.41 (*SD* = 2.94). All participants were compensated for their participation (25€).

### Stimuli

All the pictures used in the present study came from the Gegenfurtner et al. study [11]. Knowing that the application of the Remember/Know paradigm (see *Procedure* below) would necessarily lengthen the duration of the recognition phase of our experiment, we avoided this by reducing the number of presented pictures: we used 36 in the present study in comparison with the 48 used in the Gegenfurtner et al.) study [11]. These pictures were classified into three categories: green landscapes with fields and trees, flowers, and rock formations. For each participant, 36 pictures (12 per category) were randomly chosen from a database of 72 pictures (24 per category) and were used as target pictures. Half of these 36 pictures (6 per category) were randomly chosen to be presented in color while the other half were presented in black and white. The remaining 36 pictures were used as distractors at the recognition phase. In each group pictures presented in color to one half of the participants were presented in black-and-white to the other half of the participants and vice versa. The photometric luminance component of the pictures was measured and was identical for both the colored and the black-and-white pictures; the space-averaged mean luminance was approximately 35 cd/m^2^.

### Procedure

The procedure, adapted from Gegenfurtner et al. [11], consisted of two phases: an encoding phase during which participants were sequentially presented with a set of 36 pictures, and a recognition phase. Before starting the encoding phase, participants were told that they were going to see some pictures and that their recognition of these pictures would be tested later. At the encoding phase, each of the 36 pictures was presented for 1000 ms, with a 5-s interval between successive pictures. Each picture was followed by the presentation of a mask consisting of randomly chosen colored pixel blocks for color pictures and black-and-white blocks for black-and-white pictures. The mask appeared for 200 ms, followed by a uniform gray field. Pictures that were presented in color (or black and white) during the encoding phase were always presented in color (or black and white) at the recognition phase. Immediately after the encoding phase ended, the experimenter provided instructions for the recognition task. The 36 target pictures were randomly intermixed with 36 new pictures. The participants’ task was to indicate whether the seen picture had been presented or not at the encoding phase. The recognition phase was self-paced and each picture was presented until the participant gave a response by pressing a computer key. A recently developed procedure mixing confidence and Remember/Know decisions was adopted [14]. Participants were instructed to push the “1” key if they were sure that the image was new, the “2” key if they thought that the item was probably new, and the “3” key if they guessed that the item was new. They were then told to push the “8” or the “9” key if they were sure that the image was old, the key “6” or “7” if they thought that the image was probably old, and the “4” and “5” key if they guessed the item was old. Finally, the difference between the R (i.e., 4, 6 and 8) and K (i.e., 5, 7 and 9) responses was explained. Participants were instructed to use the R response if they could remember a specific event that occurred when that picture was presented in the first phase of the experiment. This event could be a thought or a feeling, for instance, they might remember having thought of their last holiday in Greece through seeing that picture, or having felt amused while seeing it. The event could also be something that occurred in the environment, such as a noise in the corridor or a flickering on the screen perceived while they were seeing that picture. Participants were also told that recognition is not necessarily associated with a remembering experience. Instead, a picture may just seem familiar to them but they think that the picture is one they saw in the encoding phase. Participants were asked to use the K response when recognition was associated with a feeling of familiarity in the absence of recollective experience. A summary of the instructions was available on a sheet of paper throughout the experiment. Participants were also instructed to justify every Remember response, i.e. to explain why they thought experiencing a recognition based on remembering. These comments were recorded and later transcribed. The amount of time needed to provide these instructions varied from 3 to 5 min across the participants. This time period was not significantly different between the two groups, *t*<1.

At both the encoding and the recognition phases, stimuli were presented on a 17-inch monitor controlled by a PC and were viewed at a distance of approximately 60 cm. The size of pictures on the screen was 512×768 pixels. The E-Prime 1.0 Software was used to present the stimuli and to record the participants’ responses and confidence ratings.

## Results

An alpha level of .05 was set for all the statistical tests. Descriptive data are presented in Table 1.

**Table 1:** Mean scores of accuracy (Hits, Hits-FAs), discrimination (*A’*), bias (*B”D*), response confidence, proportions of Remember hits, and proportions of Remember responses based on color information as a function of the group of participants and the mode of presentation.

Group	Dichromats		Normal trichromats		
*Presentation*	*Color*	*Black-and-white*	*Color*	*Black-and-white*	
Hits	0.537 (0.159)	0.444 (0.135)	0.540 (0.202)	0.434 (0.153)	*
Hits-FAs	0.461 (0.180)	0.361 (0.165)	0.464 (0.222)	0.321 (0.152)	*
*A’*	0.832 (0.087)	0.795 (0.087)	0.834 (0.089)	0.775 (0.076)	*
*B”D*	0.373 (0.214)	0.479 (0.182)	0.355 (0.279)	0.464 (0.214)	*
Confidence	2.458 (0.641)	2.083 (0.732)	2.341 (0.762)	2.023 (0.523)	*
p(R/R+K)	0.552 (0.252)	0.500 (0.250)	0.513 (0.313)	0.482 (0.228)	*
R based on C	0.214 (0.260)	0.140 (0.288)	0.140 (0.288)	0.065 (0.187)	* ^,^ #

Standard deviations are shown into parentheses.

* = main effect of the mode of presentation;

# = interaction between the group and the mode of presentation.

### Accuracy

The corrected recognition performance (hits - FAs) was calculated for each participant. Guess responses (responses 4 or 5, see *Procedure* above) were excluded for calculating the rates of hits and FAs. Indeed, previous literature has suggested that Guess responses do not reflect memory for the items that elicit these responses [12], [15]. A two-way 2 (Group: NTs *vs* Dichromats) X 2 (Presentation mode: Color *vs* Black-and-white images) ANOVA with repeated measures on the last factor was conducted on the corrected performance. This analysis indicated a main effect of the mode of presentation, *F*(1,44) = 14.80, *p*<.001, *η^2^_p_* = 0.25, but no main effect of the group, *F*<1, and no interaction, *F*<1. In order to allow a direct comparison between the present study and the one by Gegenfurtner et al. [11], we conducted the same ANOVA on the rates of hits. This analysis showed the same pattern of results, i.e. a main effect of the mode of presentation, *F*(1,44) = 11.07, *p*<.01, *η^2^_p_* = 0.20, but no main effect of the group, *F*<1, and no interaction *F*<1. These analyses showed that the two measures of accuracy (corrected and uncorrected) were significantly higher for the colored than for the black-and-white images, whatever the group.

Rates of corrected recognition were also submitted to a three-way 2 (Group: NTs vs Dichromats) X 3 (Category of picture: Green landscapes/Flowers/Rock formations) X 2 (Presentation mode) ANOVA. This analysis revealed a main effect of the category, *F*(2,88) = 13.78, *p*<.001, *η^2^_p_* = 0.24, a Category X Group interaction, *F*(2,88) = 3.14, *p*<.05, *η^2^_p_* = 0.07, and a main effect of the mode of presentation, *F*(1,44) = 15.81, *p*<.001, *η^2^_p_* = 0.26. No other main effect or interaction approached significance, all *p*s>.20. Newmann-Keuls post-hoc analyses of the Category X Group interaction indicated that there was no significant difference between groups for any category of pictures. Planned comparisons showed that in NTs the recognition performance was better for rocks (*M* = 0.47, *SD* = 0.21) than for green landscapes (*M* = 0.34, *SD* = 0.22). There was no significant difference between scenes of flowers (*M* = 0.39, *SD* = 0.20) and the other categories. The pattern of results was slightly different for dichromats: their recognition performance was better for rocks (*M* = 0.55, *SD* = 0.19) than for both green landscapes (*M* = 0.38, *SD* = 0.18) and flowers (*M* = 0.31, *SD* = 0.18) with no significant difference between green landscapes and flowers.

### Discrimination and Bias

*A’* was used as a measure of discrimination, and *B”D* as a measure of bias [16]. *A’* values are known to range between 0 and 1. The higher the *A’* value, the higher the participant’s discrimination, with 0.50 representing the chance level. The *B”D* value ranges between −1 and +1. Positive values reflect a conservative bias. A more conservative bias indicates less willingness to judge images as old. The ANOVA carried out on *A’* revealed a main effect of the mode of presentation, *F*(1,44) = 10.60, *p*<.01, *η^2^_p_* = 0.19, but no main effect of the group, *F*<1, and no interaction *F*<1. Discrimination was significantly better for colored than for black-and-white images, whatever the group. The ANOVA carried out on *B”D* showed a main effect of the mode of presentation, *F*(1,44) = 7.56, *p*<.01, *η^2^_p_* = 0.15, but no main effect of the group, *F*<1, and no interaction *F*<1. This analysis indicated that participants showed a significantly more conservative bias for black-and-white than for colored images independently of the group.

### Confidence

Each participant’s median confidence rating for correct positive responses (i.e., Hits + “yes” Guess responses) was separately calculated for colored and for black-and-white images. In order to carry out this analysis, it was considered that “8” and “9” responses corresponded to a confidence level of 3 (sure old), “6” and “7” responses corresponded to a confidence level of 2 (probably old) and that “4” and “5” responses corresponded to a confidence level of 1 (guess old). A Group X Presentation mode ANOVA revealed a main effect of the mode of presentation, *F*(1,44) = 7.73, *p*<.01, *η^2^_p_* = 0.15, but no main effect of the group, *F*<1, and no interaction *F*<1. Participants’ confidence was higher for colored than for black-and-white images, whatever the group.

### State of Consciousness Associated with Recognition

A Group X Presentation mode ANOVA was conducted on the proportions of Remember responses among the hits, i.e. R/R+K. No main effect of the mode of presentation, no main effect of the group and no interaction approached significance, all *F*s<1.5, all *p*s>.20.

Participants’ justifications for all their Remember responses were analyzed. More precisely, the proportion of justifications that included a reference to color (including black and white) was calculated for each participant. Examples of such justifications were “I remember that one, and I am sure because of the strong carmine red color of the flowers” or “Sure that I have seen it, I remember making an effort to memorize these colors, orange and mauve.” Two coders checked the occurrence of justifications that included a reference to color. A Cohen’s kappa was calculated to estimate the inter-coders’ agreement. The value of this coefficient was equal to 0.89, which represents an excellent agreement [17].

The percentage of correct Remember responses based on color information was calculated for each participant. A Group X Presentation mode ANOVA was carried out on these percentages showed no main effect of the group, *F*<1, but a main effect of the mode of presentation, *F*(1, 40) = 18.36, *p*<.001, *η^2^_p_* = 0.32, and an interaction between the two factors, *F*(1, 40) = 7.42, *p*<.01, *η^2^_p_* = 0.16. The degrees of freedom of this analysis are different from that of preceding analyses due to the fact that some participants did not give any Remember response for at least one of the two presentation modes. Newmann-Keuls post-hoc tests indicated that the proportions of justifications referring to color information were more frequent in NTs than in dichromats for colored images, but that there was no significant difference between the two groups for the black-and-white images. Planned comparisons showed that references to color in justifications were more frequent for colored than for black-and-white images in NTs, but that there was no significant difference between the two modes of presentation in dichromats.

The following colors were mentioned in NTs’ justifications for Remember responses to colored pictures (the number of Remember responses involved is given into parentheses): mauve (7), red (7), blue (6), white (6), yellow (4), orange (3), black (2), green (1), gray (1) and coppery (1). Colors mentioned in the justifications for Remember responses to black-and-white pictures were black (3), white (1) and yellow (1). In their justifications for Remember responses to colored pictures, deuteranopes referred to red (5), black (3), white (2), yellow (2) and mauve (1). For responses to black-and-white pictures, deuteranopes referred to black (4), white (3), blue (1) and yellow (1). Finally, when justifying Remember responses to colored pictures, protanopes referred to blue (2), white (2), red (1), green (1) and yellow (1). No color was specified in the protanopes’ justifications for Remember responses to black-and-white images. Note that several colors could be cited in one justification and that some participants said that color was the feature on which they based their Remember responses, without specifying which color it was.

## Discussion

The aim of the present study was to evaluate whether normal and dichromat observers differ in their memory for colored and black-and-white natural scenes, and more specifically to assess whether dichromats’ performance is enhanced by color to the same extent as is normal trichromats’ (NTs) performance. Gegentfurtner et al.’s [11] previous study addressed that point and showed that color enhanced recognition accuracy to the same extent in dichromats as in NTs. Results of the present study replicated Gegentfurtner et al.’s [11] results: both dichromats’ and NTs’ recognition accuracy (Hits) was better for colored than for black-and-white images, and the magnitude of the advantage for colored images did not significantly differ across groups. In addition, it was shown here that this pattern of results remained even when the dependent measure was the corrected performance (Hits-FAs).

In order to compare dichromats’ and NTs’ recognition memory performance in a more complete way, several other parameters were considered in the present study: discrimination, bias, confidence ratings and state of consciousness associated with recognition. Results indicated that both dichromats and NTs showed better discrimination scores and lower bias scores for colored than for black-and-white images, the difference between colored and black-and-white images being similar in both groups. In both dichromats and NTs, confidence ratings were higher for colored than for black-and-white images; the advantage for colored images did not differ significantly between the two groups. However, in both groups again, the rates of Remember responses among hits were not higher for colored than for black-and-white images.

However, the analysis of justifications for Remember responses showed an important difference between NTs and dichromats. NTs used color information to justify their Remember responses significantly more often than did dichromats, and this difference occurred only for colored images, not for black-and-white ones. In addition, NTs’ Remember responses were more often based on color information for colored than for black-and-white images whereas there was no difference between the two kinds of images in dichromats. Taken together, these results indicate that even though the rates of Remember responses were similar in NTs and dichromats, color information was underused by dichromats compared with NTs, especially when remembering colored images. This underuse might be the direct consequence of dichromats’ perceptual difficulties or the result of a more indirect influence of dichromats’ metacognitive knowledge that their color vision is deficient. Indeed, it is possible that dichromats might avoid relying on color information because they know that they may be wrong. The present study did not produce data that could help in evaluating the respective role of purely perceptual and metacognitive factors. However our study suggests that when memorizing complex natural scenes, dichromats (as well as NTs in many trials too) used a variety of other available pieces of information (e.g., the shape, the size, the position or the clarity of elements in an image), allowing them to reach the same rate of conscious recollection as NTs.

In conclusion, the present study showed that despite their reduced color discrimination, color deficient people’s memory for visual scenes is not impaired. Recognition accuracy, discrimination, bias and confidence were influenced by color in the same way in people with color deficiency as in normal observers. However the present study also indicates that color information plays a less important role in the conscious recollection of colored scenes in dichromats than in normal observers. This result indicates that it would be premature to conclude that color vision deficiency has no influence at all on recognition memory of visual scenes.
