# Correction: Recognition Memory for Colored and Black-and-White Scenes in Normal and Color Deficient Observers (Dichromats)

**DOI:** 10.1371/journal.pone.0102931

**Published:** 2014-07-10

**Authors:** The PLOS ONE Staff

There are errors in Table 1. Please see the corrected Table 1 here.

**Table 1:** Mean scores of accuracy (Hits, Hits-FAs), discrimination (*A’*), bias (*B”D*), response confidence, proportions of Remember hits, and proportions of Remember responses based on color information as a function of the group of participants and the mode of presentation.

Group	Dichromats		Normal trichromats		
*Presentation*	*Color*	*Black-and-white*	*Color*	*Black-and-white*	
Hits	0.537 (0.159)	0.444 (0.135)	0.540 (0.202)	0.434 (0.153)	*
Hits-FAs	0.461 (0.180)	0.361 (0.165)	0.464 (0.222)	0.321 (0.152)	*
*A’*	0.832 (0.087)	0.795 (0.087)	0.834 (0.089)	0.775 (0.076)	*
*B”D*	0.373 (0.214)	0.479 (0.182)	0.355 (0.279)	0.464 (0.214)	*
Confidence	2.458 (0.641)	2.083 (0.732)	2.341 (0.762)	2.023 (0.523)	*
p(R/R+K)	0.552 (0.252)	0.500 (0.250)	0.513 (0.313)	0.482 (0.228)	
R based on C	0.214 (0.260)	0.140 (0.288)	0.399 (0.310)	0.065 (0.187)	* ^,^ #

Standard deviations are shown into parentheses.

* =  main effect of the mode of presentation;

# =  interaction between the group and the mode of presentation.
